# A Clinical Comparative Study of Schnider and Eleveld Pharmacokinetic–Pharmacodynamic Models for Propofol Target-Controlled Infusion Sedation in Drug-Induced Sleep Endoscopy

**DOI:** 10.3390/biomedicines13040822

**Published:** 2025-03-29

**Authors:** Narcis-Valentin Tănase, Răzvan Hainăroșie, Lăcrămioara-Aurelia Brîndușe, Dan Corneci, Catalina Voiosu, Andreea Rusescu, Cristian Cobilinschi, Camelia Stanciu Găvan, Viorel Zainea

**Affiliations:** 1 Department of Anaesthesia and Intensive Care Medicine, Carol Davila University of Medicine and Pharmacy, 050474 Bucharest, Romania; dan.corneci@umfcd.ro (D.C.); cristian.cobilinschi@umfcd.ro (C.C.); 2 Clinic of Anaesthesia and Intensive Care Medicine, Dr. Carol Davila Central University and Emergency Military Hospital, 010825 Bucharest, Romania; 3 Department of E.N.T., Institute of Phonoaudiology and Functional Surgery Prof. Dr. D. Hociota, Carol Davila University of Medicine and Pharmacy, 050474 Bucharest, Romania; razvan.hainarosie@umfcd.ro (R.H.); catalina.pietrosanu@umfcd.ro (C.V.); andreea.rusescu@umfcd.ro (A.R.); viorel.zaine@umfcd.ro (V.Z.); 4 Department of Public Health and Management, Carol Davila University of Medicine and Pharmacy, 050463 Bucharest, Romania; 5 Clinic of Anesthesiology and Intensive Care, Clinical Emergency Hospital, 014461 Bucharest, Romania; 6 Department of Thoracic Surgery, Dr. Carol Davila Central University and Emergency Military Hospital, 010825 Bucharest, Romania; camigavan@gmail.com

**Keywords:** obstructive sleep apnea, pharmacokinetics, drug-induced sleep endoscopy, target-controlled infusion, bispectral index, Eleveld model, Schnider model, sedation techniques

## Abstract

**Background:** Optimizing sedative techniques for drug-induced sleep endoscopy (DISE) enhances accuracy and reproducibility in tailoring treatment for obstructive sleep apnea (OSA). The Schnider and Eleveld pharmacokinetic–pharmacodynamic (PK-PD) models, which predict propofol concentration in effect-site compartment based on patient-specific parameters, were utilized to guide intravenous sedation in this study. We compared the effectiveness of propofol sedation guided by the novel general-purpose Eleveld model versus the Schnider model using target-controlled infusion (TCI) systems. **Methods:** We investigated twenty-five adult OSA patients, randomized into two groups: the Schnider model group (*n* = 12) and the Eleveld model group (*n* = 13). DISE was conducted following standardized protocols, targeting effect-site concentration TCI mode. Data concerning sedation levels, effect-site concentration of propofol, procedural timing, propofol dosages, respiratory and cardiovascular parameters, and any procedural incidents were collected. **Results:** DISE was performed successfully in all enrolled patients from both groups. A significant difference was observed in the effect-site concentration of propofol (CeP) at the moment of endoscopy between the Eleveld and Schnider groups (2.1 ± 0.4 µg/mL vs. 3.3 ± 0.7 µg/mL, respectively; *p* < 0.001). The E group also demonstrated a shorter time to attain the optimal sedation plane compared to the S group (6.1 ± 1.7 vs. 9.8 ± 2.2 min, respectively; *p* < 0.001) and a reduced total procedural time (11.2 ± 1.4 vs. 15.0 ± 2.1 min, respectively; *p* < 0.001). The incidence of adverse events was comparable between groups. **Conclusions:** The Eleveld model demonstrated a shorter time to achieve the optimal sedation plane, a shorter total procedural time, and a significant difference in effect-site concentration at the time of endoscopy compared to the Schnider model. The incidence of adverse events was comparable between the two groups, suggesting that the Eleveld model may offer improved efficiency without compromising safety during DISE.

## 1. Introduction

Obstructive sleep apnea (OSA) is one of the most common sleep-related breathing disorders [1], marked by recurrent repeated partial or complete obstructions of the upper airway, leading to repeated breathing pauses in breathing, resulting hypoxemia, and disruption of normal sleep architecture [2]. With a global estimated prevalence of approximately 22.6% [3], OSA induces multiple serious chronic clinical consequences, especially when it remains undiagnosed and untreated [1,4], emphasizing the paramount importance of accurately defining and treating this very heterogeneous condition. The underlying mechanism of the airway collapse remains incompletely understood and the main pathophysiological characteristics of OSA reside in intermittent episodes of hypoxemia and sometimes hypercapnia, induced by the repetitive breathing pauses [5]. During sleep, a reduction in pharyngeal muscle tone predisposes and leads to a partial or complete collapse of the upper airway, particularly in individuals with anatomically narrow airways, enlarged soft tissue structures of the neck, and craniofacial abnormalities. The airway collapse induces airflow obstruction despite preserved respiratory effort and this obstruction predisposes to reduction in oxygen saturation and brief arousals during sleep (to recover airway patency), creating a cycle of disrupted sleep architecture. Consequently, these events contribute to oxidative stress, systemic inflammation, and increased cardiovascular and metabolic risk.

Although the majority of OSA patients respond effectively to CPAP therapy, a subset may require alternative treatments [6], including different site surgical interventions, oral appliances, myofunctional therapy, or hypoglossal stimulation techniques [7]. Drug-induced sleep endoscopy (DISE) is considered the appropriate investigation that enables the clinician to identify and reliably evaluate the airway obstruction sites and their dynamic pattern during pharmacologic-induced sleep [7,8,9]. During the procedure, the surgeon uses a flexible endoscope to examine the airway once the patient reaches the desired level of sedation and exhibits snoring or apneic events. DISE has emerged as the preferred diagnosis tool for evaluating the upper airway in this condition. Although guided mainly by expert opinion, recommendations regarding indications and technical aspects of DISE have been very recently elaborated in a European Consensus statement [10]. DISE is not considered essential in *all* sleep surgery cases but is essential prior to performing sleep surgery on the palate, in multi-level surgery, and in deciding the type of surgery [10].

Since the goal of DISE is to assess the obstruction patterns in OSA, its accuracy relies implicitly on replicating the upper airway (UA) physiological changes characteristic of natural sleep. Propofol, the most extensively studied sedative agent in this context, exhibits certain similarities to the UA physiology observed during the transition to unconsciousness in natural sleep [11]. Research involving propofol, midazolam, or their combination has demonstrated validity, along with moderate to good test–retest and inter-rater reliability for DISE findings [12].

The latest European position paper on DISE in adults, published in 2018 [13], an update of the first consensus from 2014 [14], recommends target-controlled infusion (TCI) techniques as the first option in delivering propofol sedation for DISE. The cited consensuses do not propose a specific pharmacokinetic–pharmacodynamic (PK-PD) model for propofol administration. TCI sedation offers the benefit of maintaining a more stable and controlled level of sedation, as increasing propofol concentrations correlate with greater pharyngeal muscle relaxation and increased collapsibility in a dose-dependent manner [15]; consequently, precise sedation control is critical in this setting. Additionally, it is well established in the scientific literature that sedation protocols for DISE have not yet been standardized [1,13,16] and, taking into consideration the determinant role of DISE in establishing the collapse site and obstructive dynamic pattern [17], efforts should be made in optimizing sedation in this perspective.

The Marsh (*n* = 20) [18] or the Schnider (*n* = 24) [19] PK-PD models are employed to the same extent in different studies addressing optimal sedation for DISE [20,21,22]. The Schnider model is a widely validated three-compartment PK-PD model that predicts propofol concentration based on patient-specific covariates such as age, weight, height, and lean body mass, providing effective dosage guidance in routine clinical sedation and anesthesia. Recently, Eleveld described a new PK-PD model for propofol for a broad population range [23], developed to address limitations in previous models and improve accuracy in predicting drug concentrations. This “general-purpose” model presents multiple advantages in reducing the risk of incorrect selection of a specific model for a particular patient, extrapolation, and unfamiliarity of clinician anesthesiologists with preexisting models [24]. TCI systems represent a software-guided method for drug administration of intravenous drugs. The initial bolus dose and the succeeding delivery rate of a specific drug are adapted in conformity to population-derived PK-PD models [25]. PK-PD models based on intravenous sedation have become recognized as essential tools in optimizing sedative dosing for DISE [10,13,14], providing critical insights to ensure both effective and stable plane of sedation and patient safety during airway evaluation. Considering that the prevalence of obesity among patients with OSA (recently described as being as high as 39% in patients with any OSA and 54% with severe OSA) [26] and that obesity has an important multifaceted pathogenic role in OSA, especially at high body mass index [27], selecting an appropriate PK-PD model for propofol delivery in order to account for the pharmacokinetic and pharmacodynamic characteristics specific to this patient category is of crucial importance for obese OSA patients.

Moreover, conducting DISE at the lowest minimal effective effect-site propofol concentration could mitigate the drug’s impact on the UA dynamics, preserving its function during pharmacologically induced sleep. An increase in airway collapsibility is attributed to a propofol dose-dependent inhibition of genioglossus muscle activity, likely resulting from a combination of suppressed upper airway reflexes and reduced central respiratory output to the airway dilator muscles [15].

The Marsh and Schnider PK-PD models are equally utilized in clinical practice for providing sedation for DISE, and to our best knowledge, the Eleveld model has yet to be tested in this setting. The new general-purpose models probably have the potential to enhance the clinical adoption of TCI, as they impose fewer limitations on patient selection compared to the “older” available commercial models [25].

In this study, we aimed to investigate from a clinical perspective the new PK-PD general-purpose model Eleveld in the context of propofol TCI sedation for DISE in OSA patients. The primary outcome was to assess the clinical comparative effectiveness of the two PK-PD models, Schnider and Eleveld, for the specified indication. The aim was to compare the effect-site concentration of propofol (CeP) delivered with the two models at the moment when pharmacodynamically, the patients attain the desired plane of sedation (i.e., measured bispectral index ranging between 65 and 75), enabling the endoscopic airway evaluation, and also to compare the propofol dosages required for this procedure.

Secondary outcomes included the comparative assessment of the occurrence of respiratory depression during the procedure, sedation depth objectively measured using the bispectral index (BIS), the occurrence of sneezing and cough reflex, the time elapsed from the start of sedation until the endoscopic procedure commencement, the total procedure time, and the incidence of cardiovascular adverse events such as hypotension and bradycardia.

In summary, this research aims to respond to the question of whether the novel Eleveld PK-PD model can offer benefits in terms of more accurate, safer, and shorter TCI sedation procedures for DISE in OSA patients. The Eleveld model has been relatively underexplored in the context of clinical sedation, and this study aims to provide some insights into its potential role or benefits. As we already mentioned, performing the endoscopy at the lowest effect-site propofol concentration offers advantages in the accuracy of data collected during the endoscopic evaluation (e.g., anatomical sites and degree of obstruction) and consequently optimizes the surgical decision for OSA.

## 2. Materials and Methods

### 2.1. Study Design

This pragmatic prospective randomized study was conducted at Central University and Emergency Military Hospital “Dr. Carol Davila” (tertiary teaching hospital) and Ria Clinic Cotroceni (expertise referral center for OSA) in Bucharest between March 2023 and July 2024. The Institutional Ethics Committee approved this study in February 2023 (Approval Number CE-1456, Date: 13 February 2023), and it was registered in the Research Registry with the unique identifying number *researchregistry* 10706.

### 2.2. Study Population and Inclusion and Exclusion Criteria

All participants signed an informed consent form before participating in this study. We assessed 32 patients proposed for DISE for eligibility and enrolled 25 patients, who met the inclusion criteria—age between 18 and 70 at the moment of inclusion in this study; American Society of Anesthesiologists (ASA) physical status I or II; presence of documented diagnosis of mild (AHI 5–15 events/h), moderate (AHI 16–30 events/h), or severe OSA (>30 events/h)—established by the Academy Sleep Medicine Task Force [28]. We excluded from this study patients with chronic obstructive pulmonary disease, asthma, neurodegenerative or cerebrovascular diseases, chronic cardiac pathologies (e.g., congestive cardiac failure and ischemic heart disease), chronic kidney disease, chronic liver insufficiency, body mass index (BMI) > 40 kg/m^2^, and an allergy to propofol. We also excluded from this study patients with a history of alcohol use disorder or psychoactive drug consumption. Seven patients were excluded from this study (not meeting all the inclusion criteria—two patients; declining to participate—one patient; presence of exclusion criteria—four patients).

Furthermore, all patients underwent a thorough medical history review and a pre-investigation Ear, Nose, and Throat (ENT) evaluation, which included nasoendoscopy with the Müller maneuver and polysomnography (PSG). We also recorded every patient’s minimum SpO_2_ during natural sleep from the preprocedural PSG. All patients presented an indication of alternative treatment for OSA due either to documented intolerance to CPAP therapy or CPAP refusal. We enrolled all patients presenting inclusion criteria consecutively; patients were randomized equally between groups and assigned to the Schnider group (S), receiving sedation for DISE using the Schnider PK-PD model, and to the Eleveld group (E), in whom we employed the Eleveld PK-PD model. Participants were randomly assigned to treatment groups (Schnider or Eleveld) using block randomization with block sizes of four. A computer-generated random number sequence was used to create the allocation sequence, ensuring that assignment to the groups was unpredictable.

### 2.3. The Procedure

All endoscopic investigations were accomplished in the operating room. The patient’s positioning implied lying in the supine position with a pillow supporting the head. The head was maintained in a neutral position throughout the examination. A comfortable ambiance (low-intensity lighting and avoidance of noises) was continuously ensured during the procedure.

A peripheral vein in the left arm was used to establish intravenous access, and standard monitoring of the vital signs, including 3-channel ECG, non-invasive blood pressure at 3 min intervals, and pulse oximetry, was initiated (Dräger Infinity Delta multiparameter system, Drägerwerk AG & Co. KGaA., Lübeck, Germany). To objectively assess sedation levels, a single-use forehead sensor for bispectral analysis BIS™ Quatro Sensor (Covidien Ireland Limited, Tullamore, Ireland) connected to BIS monitoring (BIS VISTA™ Monitoring System, Aspect Medical International B.V., De Meern, The Netherlands) was attached. After recording a baseline BIS measurement, TCI pumps were connected to the intravenous cannula via an octopus-type connector. We performed the sedation procedure with two types of TCI system pumps: Perfusor Space TCI Syringe Pump (B. Braun, Melsungen AG, Germany) for patients randomized in group S and Alaris PK-Nexus (Becton-Dickinson, Eysins, Switzerland) for patients randomized in group E. No premedication was given, and no preparatory measures, including topical local anesthesia, nasal decongestants, or antisecretory agents, were employed.

### 2.4. Sedation Procedure and Data Collection

The sedation in the S group was performed using 1% propofol (Fresenius Kabi, Bad Homburg, Germany) with an initial target concentration of 1.0 µg/mL with an effect-site targeted concentration in the Schnider TCI model [19]. Under careful and continuous monitoring of the patient’s level of consciousness, respiratory pattern, and BIS value, the effect-site concentration was gradually increased in a stepwise manner with 0.5 µg/mL every 2 min. The goal was to achieve sedation levels between BIS 70 and 50. The patient exhibited clinical cycles of apnea and hypopnea, allowing for the otolaryngologist to successfully perform the endoscopy.

In group E, sedation was accomplished by targeting effect-site concentration with the Eleveld model [23], also setting an initial target of 1.0 µg/mL and the same 0.5 µg/mL incremental concentration increase in effect-site propofol concentration. We did not consider routine supplemental oxygen administration during DISE, but oxygen was readily available for prolonged hypoxemic episodes (SpO_2_ < 90% for >30 s).

On a pre-established paper form, we manually recorded data regarding baseline cardiovascular status (vital parameters); sedation state using BIS; time between start of sedative procedure and start of endoscopy; total time of sedative procedure; the propofol doses necessary for achieving the optimal plane of sedation (i.e., until the surgeon effectively started the endoscopic procedure); total dose of propofol required; incidence of respiratory depression (defined as SpO_2_ < 90% for >30 s); the procedural occurrence of sneezing, cough, or movement that interfered with endoscopy; and adverse cardiovascular events (hypotension, bradycardia). The upper airway was examined in a proximal-to-distal approach, from the nasopharynx to the hypopharyngeal and glottic region. All endoscopies were performed by the same experienced otolaryngologist (RH) with additional expertise in sleep medicine, assisted by at least one other specialist otolaryngologist (CV or AR). The patients were transferred to the recovery unit for vital sign monitoring following the investigation. Two types of data collection paper form were utilized during this study for every patient: the first reporting the parameters related to the sedation procedure and second reporting endoscopic details. All data on these events and outcomes were anonymized prior to analysis.

For classification purposes, the VOTE system (velum, oropharynx, tongue base, epiglottis) was used [29] for recording the sites and patterns of airway obstruction. We registered the occurrence of arterial hypotension as systolic blood pressure < 90 mmHg and bradycardia as HR < 50/min.

### 2.5. Data Analysis

The distribution of continuous variables was analyzed using the Kolmogorov–Smirnov test, and the means and standard deviation were presented. The *t*-test was used to compare the means of continuous variables between the Schnider and Eleveld groups. To compare the Q1, Q2, and Q3 quartiles of quantitative variables, box plot diagrams were used. Qualitative variables were presented as counts and percentages, and the comparation of distribution of qualitative variables between Schnider and Eleveld groups was analyzed using chi-square/Fisher’s exact test. A *p*-value under 0.05 was considered statistically significant. Data analysis was performed with SPSS 29.0 version software (Statistical Package for Social Sciences, IBM SPSS Statistics for Windows, Version 29.0. Armonk, NY, USA: IBM Corp).

Sample size estimation was based on the previous literature evaluating the Schnider and Eleveld models for propofol target-controlled infusion anesthesia [30]. We assumed as relevant for our research a mean difference of 1.0 µg/mL in effect-site propofol concentration, with estimated standard deviations of 0.66 µg/mL for the Eleveld model and 0.87 µg/mL for the Schnider model. Using an α level of 0.05 and 80% power, the estimated minimum required sample sizes were 6–8 participants for the Eleveld group and 10–12 participants for the Schnider group.

## 3. Results

A total of 25 patients were enrolled in this prospective, randomized study, comprising 12 subjects in the Schnider group (group S) and 13 in the Eleveld group (group E). We took into consideration that the relatively close number of subjects enrolled in each group mitigates any potential bias due to an imbalance in sample size. All participants successfully completed the endoscopy procedure without any complications. Statistical analyses were conducted using each group’s entire cohort of enrolled patients. Demographic characteristics, detailed in Table 1, showed no statistically significant differences between group S and group E.

There were no significant differences between the groups in terms of demographics (age, sex, height, weight, BMI), OSA severity (mean AHI and lowest SpO_2_ during natural sleep), ASA risk distribution, smoking prevalence, or the prevalence of arterial hypertension. Detailed data are presented in Table 2.

**Figure 1:**
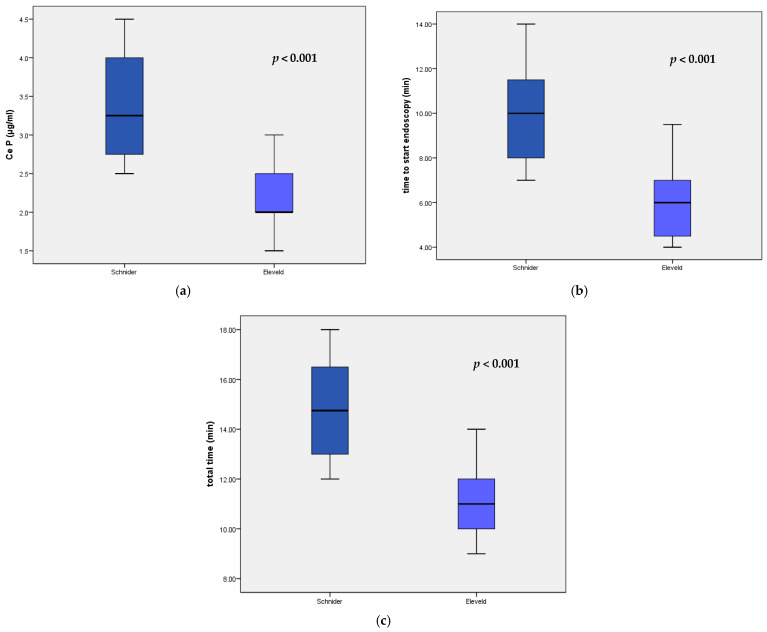
The comparative evaluation of propofol effect-site concentrations (**a**), time to reach adequate sedation state (**b**), and total time of the procedures (**c**) between the Schnider and Eleveld models.

The investigation was successfully completed in all patients. Concerning the parameters recorded during DISE, CeP was significantly lower (*p* < 0.001) in the Eleveld model group, with a mean value of 2.1 ± 0.4 µg/mL, than in the Schnider model group at 3.3 ± 0.7 µg/mL (Table 3, Figure 1).

The time required to achieve adequate sedation, measured from the initiation of sedative infusion to the successful insertion of the endoscope and commencement of the examination, was 9.8 ± 2.2 min in group S. This duration was significantly longer compared to group E, which recorded 6.1 ± 1.7 min (*p <* 0.001).

The baseline BIS (measured at the start of sedation) was similar between the two groups (*p* = 0.077). Likewise, there was no significant difference in sedation levels at the start of the endoscopy (68.8 ± 5.8 in group S vs. 66.0 ± 4.1 in group E, *p* = 0.169). Preprocedural oxygenation levels were also comparable between the groups (*p* = 0.810). Additionally, the lowest oxygen saturation observed during DISE, expressed as mean values, showed no significant difference (87.4 ± 9.6 in group S vs. 88.7 ± 6.1 in group E, *p* = 0.693). Concerning the propofol doses required to reach the adequate sedation plane, we did not register significant differences between the two groups (124.2 ± 38.7 mg in group S and 123.6 ± 36.1 mg in group E, *p* = 0.971). Significant differences in total propofol dosages necessary for completing the investigations between groups (*p* = 0.166) were not recorded.

Table 4 summarizes the incidents (i.e., cough, sneezing, or intolerance at endoscope passing) and adverse events (i.e., hypoxemia, hypotension, and bradycardia) registered in the two groups. We recorded the occurrence of cough in four patients in group S (33.3%) and in two patients (15.4%) in group E; this difference was not statistically significant (*p* = 0.294). In every group, two patients imposed supplemental oxygen administration, for severe hypoxemia (without significant difference between groups, *p* = 0.930).

The DISE findings, detailing the grade and dynamic patterns of airway obstruction, were evaluated using the VOTE classification system (velum, oropharynx, tongue base, epiglottis). Table 5 illustrates a summary of the results.

## 4. Discussion

We investigated from a clinical standpoint the utility and effectiveness of a novel general-purpose PK-PD model for propofol sedation in DISE, the Eleveld model. Several studies have confirmed the superiority of TCI technology in delivering propofol for sedation in DISE; consequently, this approach is currently recommended as a preferable practice [13,14,21]. When using an appropriate sedation technique, the endoscopist can accurately identify and assess the severity of airway obstruction, which is critical for developing personalized surgical plans for patients who cannot tolerate or refuse CPAP therapy. The Eleveld model, first described in 2018 [23] (*n* = 1033) is a comprehensive pharmacokinetic–pharmacodynamic model designed to optimize dosing by considering various patient characteristics, including age, sex, weight, organ function, and co-administration of opioids as a covariate. Specifically, it adjusts the effect-site concentration of propofol based on the presence of opioids, reflecting their synergistic effect in reducing anesthetic requirements. This enhances the model’s suitability for clinical scenarios involving opioid-based anesthesia or sedation.

Both the Schnider and Eleveld models are implemented in TCI system pumps and widely used for intravenous propofol administration. These models facilitate dosing by predicting drug concentrations in specific compartments (e.g., plasma and effect-site). In clinical practice, the Schnider model adjusts dosing based on patient characteristics like age, height, weight, and lean body mass. In contrast, the Eleveld model is validated across a wider range of populations, including pediatric, obese, and elderly patients, making it preferable for those with higher variability in pharmacokinetics. It aims to improve sedation control accuracy and was developed to address limitations in previous models, thereby enhancing accuracy in prediction of drug concentrations [31]. This general-purpose model offers advantages in minimizing the risk of incorrect model selection, extrapolation, and clinician unfamiliarity with preexisting models [24]. For DISE with propofol, both Marsh and Schnider models are equally utilized in different studies, as well as different manually controlled infusion protocols. Furthermore, the potential advantages or risks of co-sedatives associated with propofol were not addressed in the cited European consensus [13]. In a previous study [32], we explored the benefits of adding remifentanil (using the PK-PD model Minto) to a TCI-based sedation regimen with the Schnider model and highlighted the disadvantages of this common PK-PD model in obese patients.

In this pragmatic prospective randomized study, the Eleveld model for propofol sedation guided by targeting effect-site concentration was shown to be as efficient and safe as the Schnider model. Both models achieved and maintained the desired clinical level of sedation, enabling successful investigations in all patients. The incidence of noted adverse events was similar between the two groups.

While this study primarily focused on comparing the performance of the two PK-PD models in achieving adequate sedative state, the significantly lower effect-site concentration (2.1 µg/mL vs. 3.3 µg/mL) observed with the Eleveld model suggests several potential clinical advantages. This lower concentration may reduce the risk of propofol-related adverse events, including respiratory depression, and may facilitate a more precise assessment of airway dynamics during DISE.

However, the significant difference detected in effect-site concentrations (CePs) at the moment of starting the endoscopy warrants some interpretation. These differences arise from the intrinsic conceptual dissimilarities between the two PK-PD models. The Eleveld model incorporates variable volumes of distribution (V1—central compartment; V2—rapid peripheral compartment; V3—slow peripheral compartment) and employs allometric scaling for predicting these volumes. Allometry facilitates the integration of size-related covariates, allowing for biologically plausible extrapolation across diverse populations, proving particularly valuable for characterizing individuals at the extremes of the demographic spectrum [25]. In contrast, the Schnider model [19] is a three-compartment model based on a classical body size scale derived from the James equation for lean body mass. CeP is primarily influenced by the initial volume of distribution, V1 (i.e., in models with smaller V1, CeP is achieved more rapidly), as well as the drug equilibration rate constant (*k_e_*_0_) of the selected model [33]. While the *k_e0_* in the Schnider model (0.456 min^−1^) is constant, the *k_e_*_0_ in the Eleveld model is variable and dynamic, allowing for a more precise onset and offset of propofol’s effects. This enhances control during procedures where subtle titration is preferable, such as DISE, thereby reducing the risk of over- or underdosing. Formally characterized by a high V1 and a low *k_e_*_0_ (in contrast with Schnider, which features a lower V1 and a higher *k_e_*_0_), the Eleveld model could potentially predispose to an increased risk of propofol overdosing during induction, with undesired clinical consequences [25,33,34]. The extent of this plasma concentration overshoot, designed to achieve faster equilibration without exceeding the target effect-site concentration, depends on the pharmacokinetic model and, critically, on *k_e_*_0_. PK-PD models with a larger V1 and lower *k_e_*_0_ require a greater plasma concentration overshoot to achieve this gradient, compared to models with a smaller V1 and higher *k_e_*_0_. Consequently, this larger overshoot increases the risk of propofol overdose during induction, potentially leading to adverse clinical outcomes [33].

Our findings align with those of a retrospective observational study published by Linassi et al. [30], which described a similar discrepancy between CePs attained with the aforementioned two models studied during general anesthesia on adults and elderly patients. The lower propofol concentrations required in the Eleveld group may be attributed to the model’s improved ability to individualize dosing based on patient-specific factors such as age, weight, height, and lean body mass. From a clinical perspective, this is particularly relevant for obese patients with OSA, as their altered pharmacokinetics (e.g., increased volume of distribution and changes in drug clearance) can make them more susceptible to propofol overdose. By maintaining lower propofol concentrations, the Eleveld model may mitigate the risk of excessive pharyngeal muscle relaxation, which can exacerbate airway obstruction during DISE.

Furthermore, it is important to emphasize that switching to the Eleveld model from the Schnider model while targeting similar effect-site concentration to attain the required sedative plane for DISE may predispose patients to inadvertent overdosing, potentially increasing the risk of oversedation. Close attention should therefore be given to model selection and individualized dosing adjustments to ensure patient safety.

Considering the measured propofol plasma concentration prediction accuracy as a plausible explanation for the difference in CePs, it is essential to note that this accuracy has been reported to be slightly better with the Eleveld model compared to the Schnider or Marsh model, while all three models showed a good, similar median absolute performance error (MDAPE) [31]. In the same study [31], Hűppe et al. analyzed data from 50 patients and noted the tendency of both models to slightly overestimate the measured plasma concentration throughout anesthesia, by 0.64 µg/mL for Schnider and 0.53 µg/mL for Eleveld. Moreover, from a technical perspective, at the beginning of the study, we acknowledged the known discrepancies in delivering propofol sedation with the Schnider model across different manufacturers, based on different methodologies of *k_e_*_0_ calculation, therefore resulting in different induction doses in some patients [35]. For delivering sedation in the Schnider model, the Perfusor Space pump employed a fixed equilibration rate constant (*k_e_*_0_) of 0.456 min^−1^, whereas the Alaris PK Nexus pump calculated an individual *k_e_*_0_ resulting from a fixed time-to-peak effect (*t_peak_*) of 1.6 min. Consequently, we did not use the TCI pumps interchangeably between the groups.

Another finding from our study is that similar doses of propofol were required to achieve the desired depth of sedation (mean 124.2 ± 38.7 mg in group S and 123.6 ± 36.1 in group E, *p* = 0.971) for performing the endoscopy in both groups; however, the times taken to reach this sedation level were significantly different (mean 9.8 min in S group vs. 6.1 min in E group, *p* < 0.001). The models differ in their algorithms and in how they handle patient-specific parameters, which could lead to different concentration profiles despite similar total doses. Deeper sedation increases upper airway collapsibility during DISE evaluation, and as a consequence, performing DISE with BIS-guided propofol infusion, particularly at a BIS level of 65–75, provides an objective and reproducible approach to assessing upper airway collapsibility [36,37]. Both models maintained stable physiological parameters (e.g., blood pressure and heart rate) in this study. Severe desaturations imposing supplemental oxygen administration occurred in two patients in each study group.

In this study, most patients were overweight or obese, with a mean BMI of 30.4 kg/m^2^ in group S and 28.4 kg/m^2^ in group E. The Eleveld model previously demonstrated clinically acceptable performance in obese patients [38]; in contrast, using the Schnider model, which was conceived without incorporating the influence of obesity [39], seems reasonable as a second option compared with “second generation” TCI models (i.e., general-purpose models). We initiated DISE in both groups with a lower-than-usually recommended initial target concentration in the effect compartment (1 µg/mL) and incrementally increased it by 0.5 µg/mL every 2 min. This approach was designed to ensure a smooth induction process, enhance procedural safety, and minimize the risk of oversedation or airway obstruction unrelated to sleep breathing disorders. Additionally, the gradual increase in the effect-site concentration of propofol provided a precise and adequate observation window for the examination. While BMI can influence propofol pharmacokinetics [39], especially in the morbidly obese, we believe that the magnitude of this difference between groups is minor (only 2.0 kg/m^2^) and not statistically significant (*p* = 0.199). Therefore, it is unlikely to have substantially affected our findings. The observed differences in effect-site concentration and time to sedation are consistent with other published studies comparing these two PK-PD models [30], suggesting that model-specific factors are the primary drivers of the observed findings.

Furthermore, we adopted a gradual increase in effect-site concentration to address the known limitations of the Schnider model when applied to our predominantly obese patient population. Although the suggested effect-site concentration for initiation of propofol TCI sedation for DISE [13,14] in order to achieve a quicker sedation ranges between 2.0 and 2.5 µg/mL effect-site concentration, followed by incremental dose increases of 0.2–0.5 µg/mL every 2 min until the patient demonstrates snoring, vibrations, or apneic episodes, we consider that a more conservative approach will reduce risk of central apnea and false-positive airway evaluation results. It is also important to highlight that transitioning from the Schnider to the Eleveld model by an inexperienced practitioner while targeting the same initial CeP (i.e., 2.0–2.5 µg/mL) could lead to inadvertently high initial bolus doses. This practice may increase the risk of central apnea and hypoxemia, potentially resulting in inaccurate airway findings during DISE. The sedation levels at the start of the endoscopy, measured by BIS monitoring, were similar between groups S (68.8) and E (66.0, *p* = 0.169), aligning with the values considered appropriate in the existing literature [36,40,41]. Both regimens provided adequate sedation depth. Perhaps due to the relatively small sample size and the slow incremental effect-site propofol concentration escalation, we did not find significant differences in the incidences of adverse events and incidents during DISE procedures. To our knowledge, this is the first study to evaluate the clinical application of the Eleveld model for propofol dosing in the DISE setting.

Endoscopic findings using VOTE classification to assess upper airway obstruction revealed no significant differences between groups, suggesting that the PK-PD model does not affect diagnostic outcomes of DISE. Nevertheless, we note that these results may be influenced by the variability in OSA phenotypes, despite patient similarities detailed in Table 1, independent of the sedation protocols used.

The shorter time to sedation and overall procedure time associated with the Eleveld model may improve patient comfort and experience. While a 3.7 min reduction might seem modest, it can translate into substantial improvements in workflow efficiency in a busy clinical setting.

While a direct causal link between lower CeP and improved clinical outcomes is not definitively established by this study, due to the limited number of enrolled patients, the statistically significant reduction in effect-site concentration with the Eleveld model suggests potential advantages, including a potentially lower risk of propofol-related adverse events such as respiratory depression, and a potentially more accurate assessment of airway dynamics during DISE. However, the limited sample size of this study precludes definitive conclusions about the clinical relevance of these findings. Larger-scale studies are warranted to confirm these potential benefits.

Our study presents several limitations. The first limitation is the small number of patients enrolled. The relatively limited sample size, combined with the specific inclusion of certain patient populations (e.g., men with obesity), restricts the generalizability of our findings. Although the higher proportion of male patients in both groups reflects the general epidemiology of OSA, where the condition is more prevalent among males compared with females, we believe that the gender distribution in this research is predominantly specific to the study population (men presenting indication for DISE due to intolerance to CPAP or CPAP refusal). The limited sample size affects the generalizability of the findings by reducing the ability to capture the variability inherent in larger and more diverse OSA populations (i.e., phenotypes) and also gender-based differences. Additionally, this sample size may also not capture the potential benefits of Eleveld PK-PD in terms of preventing propofol overdose and the subsequent procedural hypoxemia.

Moreover, the low incidence of adverse events recorded during sedative procedures in our study may be underestimated, firstly due to the limited number of patients and secondly to the cautious protocol for increasing the effect-site concentration of propofol. The reduced CeP observed in the Eleveld group may decrease the risk of adverse events such as excessive upper airway collapse or hypoxemia, particularly in high-risk patients with OSA. Another limitation lies in the lack of data on the time required for complete resolution of sedation, defined as achieving an Aldrete score of 10. Additionally, all DISEs were conducted with patients in the supine position; however, collapse patterns may differ in the lateral position, and obstructions at the base of the tongue and larynx can improve when patients are turned to the lateral position [42], emphasizing that our findings only apply to the supine orientation.

This study’s design precluded a direct comparison of recovery times between groups. Fifteen patients (in both groups) proceeded directly to general anesthesia for OSA surgery after the DISE procedure, preventing assessment of propofol-specific recovery.

Our study focused on patients undergoing DISE for OSA, which may not represent all clinical scenarios requiring TCI sedation. The applicability of these findings to other clinical contexts should be approached with caution. Further research involving larger patient cohorts will be essential to validate these findings, explore clinical implications, and better evaluate the potential advantages of one PK-PD model over another, as well as to assess the application of the Eleveld model in different clinical settings, including OSA patients proposed for DISE.

## 5. Conclusions

The Eleveld PK-PD model demonstrates suitability for performing DISE, offering a reliable and precise approach to optimizing sedation and accurately assessing upper airway dynamics in OSA patients. To achieve optimal sedation for DISE, clinicians must be mindful of the differences between the Schnider and Eleveld models when transitioning between them. Standardizing sedation techniques and implementing protocols will enhance the assessment of obstruction patterns in the heterogeneous patient population with OSA, allowing for a more accurate distinction between the endotypes of this complex sleep-related breathing pathology and consequently improving treatment strategies. However, it is important to acknowledge that the relatively low statistical power of this study limits the generalizability of its findings, highlighting the necessity of further research to validate these results in larger patient populations.

## Figures and Tables

**Table 1:** Characteristics of patients by group.

Characteristic	Group S (Schnider) (*n* = 12)	Group E (Eleveld) (*n* = 13)	*p-*Value
Age (year) (mean ± SD)	45.7 ± 8.0	50.8 ± 9.7	0.160
Sex			
Male (%)	12 (100.0)	11 (84.6)	0.157
Female (%)	0 (0.0)	2 (15.4)	
Height (cm) (mean ± SD)	178.2 ± 6.4	173.4 ± 7.4	0.098
Weight (kg) (mean ± SD)	96.7 ± 14.7	85.7 ± 14.7	0.073
Body mass index (kg/m^2^) (mean ± SD)	30.4 ± 3.8	28.4 ± 3.9	0.206
Ponderal status			
Normoponderal (BMI 18.5–24.9 kg/m^2^)	1 (8.3)	2 (15.4)	0.199
Overweight (BMI 25–29.9 kg/m^2^)	4 (33.3)	8 (61.5)	
Obese (BMI > 30 kg/m^2^)	7 (58.3)	3 (23.1)	

**Table 2:** Preprocedural characteristics of patients by group.

Characteristic	Group 1 (Schnider) (*n* = 12)	Group 2 (Eleveld) (*n* = 13)	*p*-Value
Apnea–hypoxia index in PSG (mean ± SD)	43.5 ± 20.8	31.2 ± 15.7	0.106
Hypertension (%)	5 (41.7)	5 (38.5)	0.870
Smoking (%)	3 (25.0)	3 (23.1)	0.910
Lowest SpO_2_ in normal sleep (%) (mean ± SD)	76.4 ± 10.6	83.9 ± 9.4	0.073
ASA physical status I/II (%)	5 (41.7)/7 (58.3)	6 (46.2)/7 (53.8)	0.821

**Table 3:** Comparison between parameters registered during DISE.

Characteristic	Group S (Schnider) (*n* = 12)	Group E (Eleveld) (*n* = 13)	*p*-Value
Success (%)	12 (100.0)	13 (100.0)	1.000
Time to start endoscopy (min) (mean ± SD)	9.8 ± 2.2	6.1 ± 1.7	<0.001
Total time of the procedure (min) (mean ± SD)	15.0 ± 2.1	11.2 ± 1.4	<0.001
Lowest SpO_2_ during DISE (mean ± SD)	87.4 ± 9.6	88.7 ± 6.1	0.693
Preprocedural SpO_2_ (mean ± SD)	98.3 ± 1.2	98.5 ± 1.5	0.810
CeP * (mean ± SD)	3.3 ± 0.7	2.1 ± 0.4	<0.001
BIS at starting endoscopy (mean ± SD)	68.8 ± 5.8	66.0 ± 4.1	0.169
BIS baseline (mean ± SD)	97.2 ± 1.4	95.8 ± 1.8	0.077
Propofol dose (mg) until reaching adequate sedation plane	124.2 ± 38.7	123.6 ± 36.1	0.971
Propofol dose total (mg)	182.6 ± 44.9	158.3 ± 40.0	0.166

* CeP = effect-site propofol concentration.

**Table 4:** Incidents recorded during drug-induced sleep endoscopy by group.

Characteristic	Group S (Schnider) (*n* = 12)	Group E (Eleveld) (*n* = 13)	*p*-Value
Cough (%)	4 (33.3%)	2 (15.4%)	0.294
Hypoxemia (%)	2 (16.7%)	2 (15.4%)	0.930
Hypotension (%)	1 (8.3%)	2 (15.4%)	0.930
Bradycardia (%)	0 (0.0%)	1 (7.7%)	0.327

**Table 5:** Endoscopic findings, described using VOTE classification.

Characteristics of Obstruction	Schnider Group (*n* = 12)	Eleveld Group (*n* = 13)	*p-*Value
Velum			
Anteroposterior (partial/complete)	2 (16.7)/2 (16.7)	3 (23.1)/3 (23.1)	0.808
Lateral (partial/complete)	0 (0.0)/1 (8.3)	1(7.7)/0 (0.0)	0.367
Concentric (partial/complete)	2 (16.7)/5 (41.7)	2 (15.4)/4 (30.8)	0.817
Oropharynx			
Lateral (partial/complete)	5 (41.7)/3 (25.0)	7 (53.8)/1 (7.7)	0.495
Tongue base			
Anteroposterior (partial/complete)	4 (33.3)/0 (0.0)	3 (23.1)/0 (0.0)	0.673
Epiglottis			
Anteroposterior (partial/complete)	1 (8.3)/0 (0.0)	2 (15.4)/0 (0.0)	0.588
Lateral (partial/complete)	0 (0.0)/0 (0.0)	2 (15.4)/0 (0.0)	0.157

## Data Availability

Data are unavailable due to privacy restrictions. Derived data supporting the findings of this study are available from corresponding authors N.V.T. or L.A.B. on request.
